# Polyphenol and exercise-induced molecular crosstalk: a new frontier in brain function, head and neck cancer therapy, and healthy lifespan extension

**DOI:** 10.3389/fnut.2025.1624047

**Published:** 2025-09-02

**Authors:** XiuLei Mao, Sha Yang

**Affiliations:** Department of Physical Education and Health, Lyuliang University, Lvliang, Shanxi, China

**Keywords:** head cancer, laryngeal cancer, oropharyngeal cancer, dysphagia, diabetes mellitus

## Abstract

Emerging evidence highlights the synergistic potential of polyphenols and exercise in modulating molecular pathways critical for brain function, tumor suppression, and healthy aging. Both interventions independently influence oxidative stress, inflammation, apoptosis, autophagy, metabolic regulation, and neuroplasticity—key processes implicated in head and neck cancers (HNCs) development, neurodegeneration, and lifespan determination. Recent studies reveal that polyphenols, through their antioxidant and epigenetic-modifying properties, enhance neuronal resilience, suppress tumorigenesis, and improve metabolic homeostasis. Simultaneously, exercise activates signaling cascades, promoting neurogenesis, immune modulation, and anti-cancer effects. The molecular crosstalk between polyphenol intake and physical activity appears to amplify protective mechanisms, offering novel therapeutic strategies for brain health preservation, HNCs management, and metabolic optimization. This review critically examines the interconnected pathways influenced by polyphenols and exercise, their combined impact on brain function and tumor suppression, and discusses the translational potential for extending healthy lifespan through integrated lifestyle interventions.

## 1 Introduction

Head and neck cancers (HNCs) are a varied range of cancers which arise from paranasal sinuses, mouth, nose, salivary glands, pharynx, and larynx. HNCs are mainly squamous cell cancer and thus, they are mentioned to as head and neck squamous carcinomas (HNSCCs). According to global statistics about these cancers, oral and lip cancers (which are the most public types of HNSCC) have affected 377,713 new cases in 2020 and have taken the lives of 177,757 patients (1). Other than the ranking of these cancers which is significantly high among all cancers, the growing incidence of HNCs, due to increased consumption of alcohol and tobacco and HPV infection, has created some concerns in both developing and developed countries (2). Contrary to their relatively high prevalence, these cancers are not well studied and are slightly underestimated. Exercise has been acknowledged for a long time as a key element in treating various illnesses and cancer is no exception. Nonetheless, it’s crucial to assess the advantages and drawbacks of exercise programs to confirm that the benefits surpass the risks. According to a great body of research, different types of exercise including Aerobic, Resistance, Functional, Balance and other trainings such as flexibility, high-intensity interval training (HIIT) and stretching activities can be used in both individual and group forms for affecting cancer hallmarks and also enhancing the quality of life (QoL) in cancer patients who has undergone invasive therapeutic methods.

Understanding the molecular crosstalk between polyphenol intake and physical exercise suggestions a novel and promising approach to enhancing brain health, preventing and treating HNCs, managing metabolic disorders especially diabetes, and promoting healthy lifespan extension (3, 4). It was documented that both polyphenols and physical exercise independently activate main cellular signaling paths, such as PI3K/Akt/mTOR, AMPK, SIRT1, and Nrf2, which are essential for neuroprotection, metabolic regulation, inflammation control, and tumor suppression (5–8). When combined, these interventions may synergistically optimize mitochondrial function, suppress oncogenic processes, enhance autophagy, and counteract oxidative damage and neurodegeneration (9–11). Given the rising burden of HNCs, neurodegenerative diseases, and metabolic disorders in aging populations, the integration of dietary polyphenols with structured exercise interventions provides a cost-effective, non-pharmacological strategy with wide-reaching clinical applications. Moreover, this method aligns with the principles of preventive, predictive, and personalized medicine, offering possible for tailored interventions based on individual molecular profiles (12, 13).

This review highlights the urgent need for interdisciplinary research combining molecular biology, neuroscience, nutrition science, oncology, and exercise physiology to fully unlock the therapeutic potential of polyphenol-exercise synergy in promoting brain resilience, suppressing tumor progression, and extending healthspan.

## 2 HNSCC

**Figure 1:**
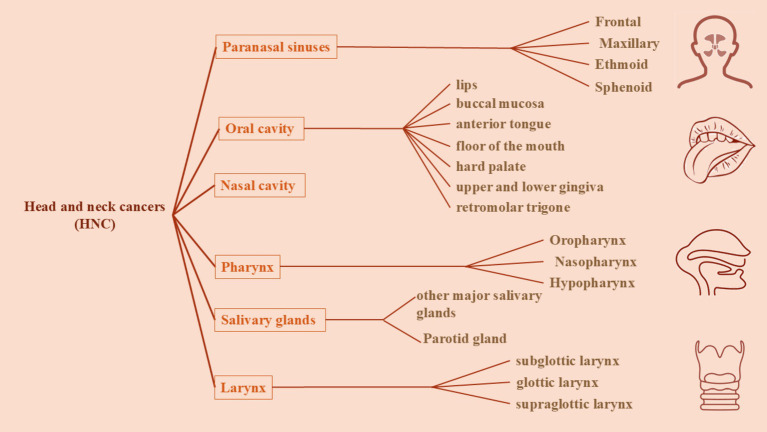
A schematic presentation of the classification of head and neck cancers based on their sites. Head and neck cancers are classically defined as neoplasms originating from specific sites in head and neck including mucosal tissues of paranasal sinuses, oral cavity (which contains lips, tongue, buccal mucosa, hard palate, and gingiva), nasal cavity, larynx (which includes supraglottic, glottic and subglottic larynx), pharynx (containing naso-, oro-, and hypo-pharynx), and salivary glands.

Head and neck squamous carcinomas are classically defined as neoplasms originating from specific sites in neck and head including mucosal tissues in paranasal sinuses, oral cavity (which contains lips, tongue, buccal mucosa, hard palate, and gingiva), nasal cavity, larynx (which includes supraglottic, glottic and subglottic larynx), pharynx (containing naso-, oro-, and hypo-pharynx), and salivary glands (potential sites of HNSCC are showed in Figure 1).

### 2.1 Epidemiology

In general, the worldwide burden of HNSCC mortality and incidence is changing. This trend is influenced by the aging and expanding population, along with shifts in the spread and prevalence of key cancer risk factors, many of which are linked to socioeconomic development. The latest study on cancer prevalence is conducted in 2020 according to the GLOBOCAN estimates of cancer mortality and incidence which is created by the International Agency for Research on Cancer (1). As reported by this study, new cases of mouth and lips cancers as well as larynx, Nasopharynx, Oropharynx, Hypopharynx, and Salivary glands are detected as 377,713, 184,615, 133,354, 98,412, 84,254, and 53,583 cases were worldwide (1). According to the statistics of this study, oral cancer is the 8th most public cancer among men while it is the 15th most public among women (1). Region-Specific Incidence of oral and lips cancers shows that Melanesia, south central Asia, eastern Europe, New Zealand and Australia are the 5 top regions for this cancer. Lip and oral cavity cancers are very common in South Central Asia (such as Sri Lanka, India, and Pakistan) and in Melanesia which shows that chewing betel nut in these regions is a vital risk factor for oral cancer (1). Over the last ten years, laryngeal cancer cases have risen by 23%. In developed countries, there has been a significant increase in cases among younger women, possibly due to evolving cultural norms regarding tobacco and alcohol use, along with a heightened burden of HPV (2). In Japan, the rates of tumors in the oral cavity, oropharynx, and salivary glands have increased further in women than in men, while the incidence of nasopharyngeal and laryngeal tumors has declined, particularly in association with certain factors (2).

### 2.2 Risk factors

**Figure 2:**
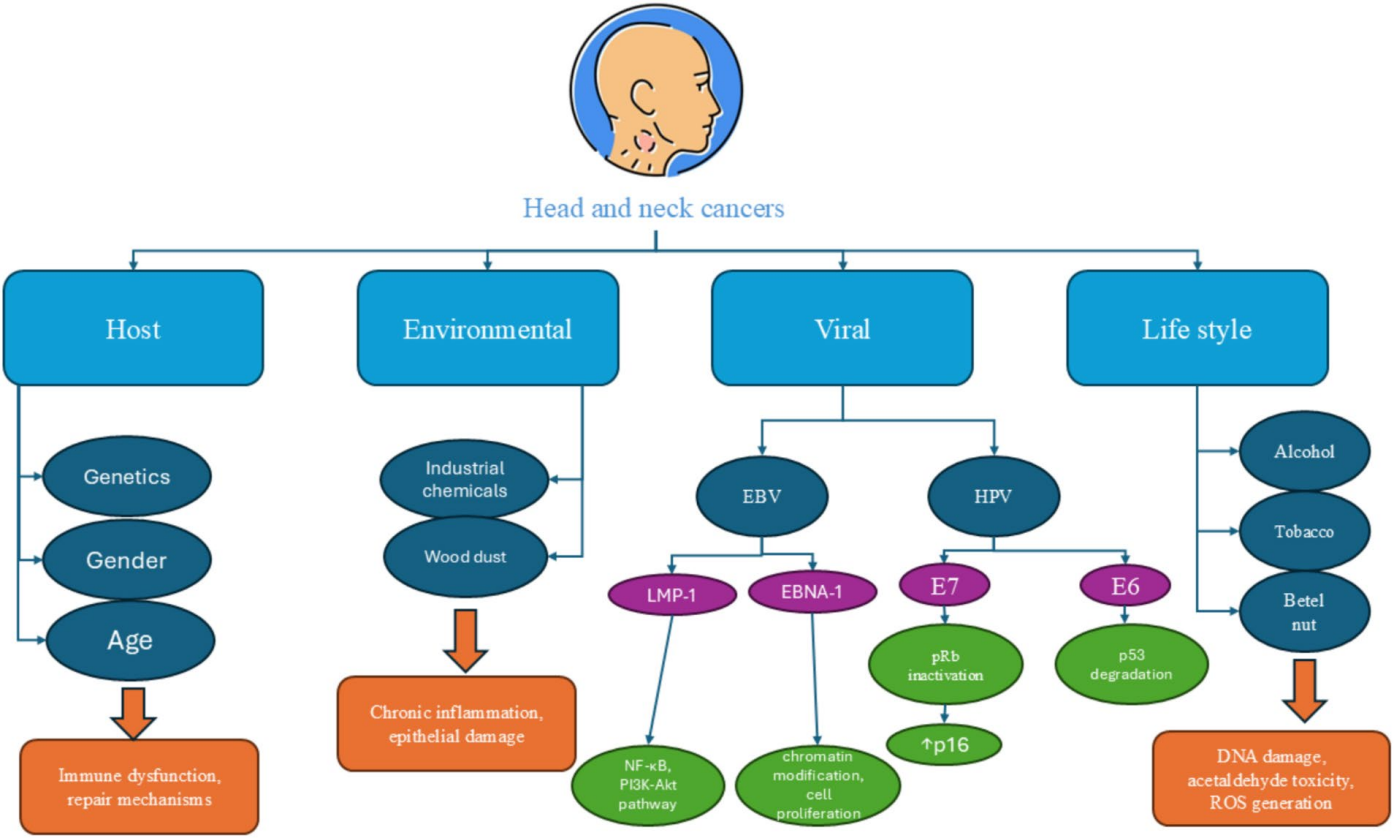
A summary of common risk factors which are involved in the pathogenesis of head and neck cancers. Alcohol consumption, tobacco use, human papilloma virus infection, and Epstein–Barr virus infection are the main risk factors for head and neck cancers.

The main risk factors typically related to HHNSCC include alcohol and tobacco use, chewing areca nut, human papillomavirus (HPV) infection, and Epstein–Barr virus (EBV) infection (particularly for nasopharyngeal cancers) (all risk factors are summarized in Figure 2).

#### 2.2.1 Tobacco use

One of the maximum significant risk factors for HNCs is tobacco use, which includes smoking cigars, cigarettes, and pipes, as well as the consumption of smokeless tobacco products (14, 15). Tobacco has many harmful chemicals linked to cancer, including nitrosamines, polycyclic aromatic hydrocarbons, aldehydes, and aromatic amines. These ingredients are produced when tobacco is burned at high temperatures and are recognized to harm the DNA in cells of the oropharynx, donating to the onset of cancer (16). Based on a study in Western Europe, tobacco consumption continues to be the primary risk factor for HHNSCC, contributing to approximately 75% of all instances; however, this data cannot be generalized and data from East Asia have suggested that tobacco use is involved in 2.8–25% of subjects (2, 14, 15). Studies have shown that individuals who smoke are six times extra likely to develop these cancers compared with non-smokers. Moreover, the risk increases with the intensity and duration of tobacco use. Even secondhand smoke exposure poses a risk, particularly to non-smokers living with smokers (14, 15).

#### 2.2.2 Alcohol consumption

Alcohol use is another main risk factor associated with HNCs. In 2012, it was reported that there were 162,547 cases of cancer affecting the oropharynx, oral cavity, and hypopharynx linked to alcohol use, which included 22,131 women and 140,416 men. The attributable fraction (AF) of alcohol use for these cases was 36.7%, with a breakdown of 44.7% for men and 17.2% for women (17). Moreover, there were 40,965 cases of laryngeal cancer linked to alcohol use, with 39,143 occurring in men and 1,821 in women. Alcohol use is responsible for 26.1% of all laryngeal cancer cases, which breaks down to 28.4% for men and 9.7% for women. The percentage of HNCs cases related to alcohol grew from 2002 to 2012 (17). Research has indicated that acetaldehyde and ethanol are the key constituents which may have cancer-causing effects in humans. Ethanol is converted into acetaldehyde through the role of the alcohol dehydrogenase enzymes (ADH), then which acetaldehyde is transformed into acetate via aldehyde dehydrogenase enzymes (ALDH). Among the ADH enzymes, ADH1B and 1C are primarily responsible for the change of ethanol to acetaldehyde, while ALDH2 is mainly responsible for converting acetaldehyde into acetate (18). To begin with, acetaldehyde interferes with repair processes and DNA synthesis and attaches to proteins, leading to changes in their structure and function. This affects key enzymes to DNA repair and methylation, along with glutathione (GSH), a crucial antioxidant peptide. Additionally, acetaldehyde can fix to DNA, leading in stable DNA adducts (19–21).

#### 2.2.3 Human papilloma virus (HPV)

Human papilloma virus is a slight DNA virus made up of double-stranded DNA and a capsid composed of two essential proteins which is sexually transmitted among humans. This virus is able to some oncoproteins when is inside a living cell (22–25). Till now, numerous types of HPV virus are detected which are classified based on the how likely it is for them to establish cancerous lesions. Low-risk types of HPV (which primarily contain HPV-6 and -11) are usually linked to non-cancerous conditions such as laryngeal papillomatosis and warts in the genital or perianal areas. In contrast, high-risk types, mainly HPV-16, −31, −18, −33, and −35 are connected to cervical cancer, certain tumors in the genital area of both women and men, and a category of HNSCC (22–25). Early protein 7 (E7) and protein 6 (E6) are two of the most important oncoproteins which are responsible for the carcinogen effects of this virus. Oncoproteins E7 and E6 play a crucial role in HPV-related tumors by inactivating the products of tumor suppressor genes pRb and p53. This disruption affects the regulatory mechanisms of the DNA repair and cell cycle pathways, which are also commonly deactivated in tumors caused via tobacco through mutations (26). In HPV-associated cancers, the E6 protein’s ability to induce the degradation of p53 is a key mechanism that contributes to tumorigenesis. By targeting p53 for degradation, HPV effectively removes a critical regulator of the cell cycle and apoptosis, resulting in unchecked cell proliferation and genetic instability (26). This disruption occurs even in the presence of wild type p53, as the viral protein can inhibit its function without necessitating mutations in the p53 gene itself. Interestingly, while some HPV-positive tumors do exhibit mutations in the p53 gene, the functional impact of these mutations can vary. Some mutations may not significantly impair p53’s ability to regulate cell growth and apoptosis, allowing these tumors to remain HPV-related despite the presence of mutations (26, 27). This complexity indicates that p53 mutational position alone is not a reliable indicator of HPV involvement in carcinogenesis. Furthermore, the reliance on p53 immunohistochemistry as a surrogate marker for p53 status can be problematic, as it may not accurately reflect the underlying genetic alterations. The labor-intensive nature of detecting p53 mutations through gene analysis also complicates the assessment of HPV’s role in tumor development (28, 29).

Also, the E7 protein of HPV serves a crucial function in the oncogenic process by inactivating the retinoblastoma (pRb) tumor suppressor protein. In addition, pRb is vital for regulating the cell cycle, and its inactivation leads to uncontrolled progression through the G1 phase, promoting cell proliferation. The relationship between E7 and pRb is well-established; however, E7 also interacts with other members of the pRb family, further contributing to its oncogenic effects (30). The E7 protein of HPV serves a crucial function in the oncogenic process by inactivating the retinoblastoma (pRb) tumor suppressor protein (31). This inactivation disrupts the regulatory control of the G1/S cell cycle checkpoint, leading to the release of E2F transcription factors and allowing uncontrolled cellular proliferation. Beyond cell cycle deregulation, pRb inactivation also contributes to a cascade of oncogenic effects, including resistance to apoptosis, increased genomic instability, and impaired cellular differentiation. These processes are especially relevant in HPV-associated head and neck cancers, where unchecked proliferation and reduced apoptosis promote tumor initiation and progression (31). Moreover, by undermining differentiation pathways, pRb inactivation fosters the maintenance of an undifferentiated, proliferative cell phenotype commonly seen in aggressive HPV-positive oropharyngeal tumors. This molecular mechanism aligns closely with key hallmarks of cancer and reinforces the central role of E7 in HPV-driven tumorigenesis (32).

As a consequence of pRb inactivation, there is an up-regulation of p16. This up-regulation occurs through a negative feedback mechanism: when pRb is inactive, it cannot suppress the expression of p16, leading to increased levels of this protein (33, 34). The presence of high p16 levels can be identified via immunohistochemistry, making it a valuable biomarker for identifying HPV-related cancers. Recent studies have suggested that tumors exhibiting HPV DNA and overexpressing p16 may represent a distinct subset of cancers that are causally linked to HPV infection. This association not only helps in identifying HPV-positive tumors but also underscores the potential for p16 as a surrogate marker in clinical settings. By using p16 expression as an indicator of HPV involvement, pathologists can better classify tumors and potentially guide treatment strategies (33, 34).

This association not only helps in identifying HPV-positive tumors but also underscores the potential for p16 as a clinically relevant surrogate marker, particularly in OPSCC. Tumors that are both HPV DNA-positive and p16-overexpressing represent a biologically and clinically distinct subset of head and neck cancers. Epidemiologically, HPV-positive OPSCCs tend to occur in younger patients with limited or no history of tobacco or alcohol use, differing significantly from the traditional demographic profile of head and neck cancer, which typically involves older, tobacco-exposed individuals. This shifting profile has led to a rise in HPV-associated cancers, especially in high-income countries, even as tobacco-related cases decline (35).

Pathologically, HPV-positive tumors often exhibit non-keratinizing morphology, basaloid features, and a high mitotic index, in contrast to the keratinizing and poorly differentiated patterns seen in HPV-negative tumors. High levels of p16 expression, used as a surrogate marker for HPV-driven oncogenesis, result from the functional inactivation of the retinoblastoma (pRb) protein by the HPV E7 oncoprotein. This loss of pRb leads to compensatory overexpression of p16, which can be detected via immunohistochemistry, offering a practical tool for clinical diagnostics (36).

Clinically, HPV-positive, p16-overexpressing OPSCCs demonstrate enhanced responsiveness to chemotherapy and radiation therapy, and patients tend to have significantly better overall and disease-free survival rates. These favorable outcomes have prompted efforts to de-escalate treatment intensity in selected patients to reduce long-term toxicity without compromising efficacy. Altogether, the distinct epidemiological, pathological, and therapeutic profiles of this tumor subset highlight the importance of accurate HPV and p16 testing, both for prognostic assessment and for informing individualized treatment strategies in clinical practice (37).

In summary, the interplay between HPV oncoproteins E6 and E7 with critical tumor suppressors like p53 and pRb, respectively, highlights the complex mechanisms through which HPV contributes to carcinogenesis. The regulation of p16 by pRb provides an additional layer of understanding regarding HPV’s role in cancer and offers a practical approach for identifying HPV-associated malignancies (22–25). These oncogenic mechanisms, specifically the inactivation of tumor suppressor proteins p53 and pRb by HPV oncoproteins E6 and E7, are most strongly implicated in the pathogenesis of HPV-positive oropharyngeal squamous cell carcinomas (OPSCC) (38). These cancers predominantly arise in the tonsillar region, the base of the tongue, and the soft palate. The presence of high-risk HPV subtypes, particularly HPV-16, is now recognized as a distinct etiological factor in these tumor types, with implications for prognosis and treatment response. In contrast, the role of HPV in cancers of the larynx, hypopharynx, and oral cavity remains less definitive and often secondary to traditional carcinogenic exposures such as tobacco and alcohol. While some studies have detected HPV DNA in a subset of these cancers, the functional significance, especially in terms of E6/E7 expression and p53/pRb inactivation, is not as clearly established as in OPSCC (38). Therefore, when discussing HPV-mediated carcinogenesis, it is critical to distinguish between tumor sites, as the molecular underpinnings and clinical behaviors differ significantly. This distinction underscores the need for site-specific diagnostic and therapeutic strategies in head and neck cancers.

Prophylactic vaccination against HPV has significantly advanced the prevention of HPV-associated diseases, including head and neck cancers, particularly OPSCC. Three main vaccines are approved: Cervarix (bivalent, targeting HPV types 16 and 18), Gardasil (quadrivalent, targeting HPV types 6, 11, 16, and 18), and Gardasil 9 (nonavalent, covering HPV types 6, 11, 16, 18, 31, 33, 45, 52, and 58) (39). These vaccines elicit a robust immune response, especially when administered prior to HPV exposure, and have demonstrated a significant reduction in the prevalence of vaccine-type HPV infections and related neoplasias. Although primarily aimed at preventing cervical and anogenital cancers, increasing evidence suggests potential benefits in reducing oral HPV infections, indicating their broader role in preventing HPV-related head and neck cancers. Public health strategies promoting early vaccination, particularly among adolescents, are essential for maximizing the protective effects of HPV immunization (39, 40).

#### 2.2.4 EBV

**Figure 3:**
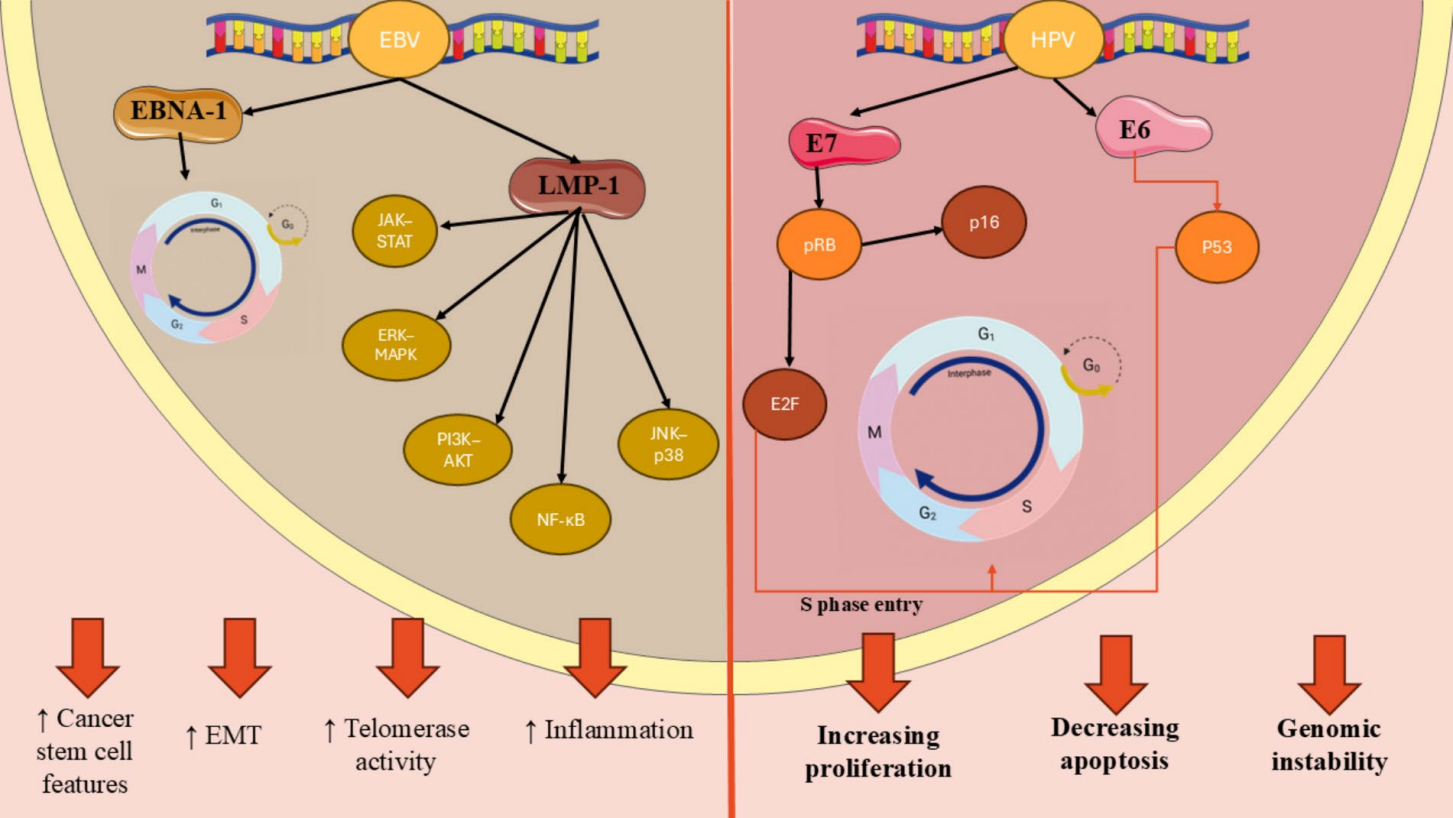
Molecular mechanisms of HPV- and EBV-induced oncogenesis in head and neck cancers. HPV E6 and E7 oncoproteins lead to p53 degradation and pRb inactivation, respectively, resulting in increased E2F activity and p16 upregulation. In contrast, EBV latent proteins such as EBNA-1 and LMP-1 activate oncogenic signaling cascades (NF-κB, PI3K–AKT, MAPK, and JAK–STAT), promoting cell proliferation, inflammation, and epithelial-mesenchymal transition.

Epstein–Barr virus is one of the most public human viruses and is recognized for its capacity to create lifelong infections (41). It primarily resides in the oral epithelium and B lymphocytes, where it can adopt two distinct phases of its lifecycle: latency and productive replication. EBV, with studies indicating that over 90% of adults worldwide have been infected with it at many point in their lives (42, 43). While many people experience mild or no symptoms during the initial infection, particularly when it occurs in childhood, EBV can lead to more significant health issues later in life like cancer. Among HNSCCs, oral cancers including oral squamous cell carcinomas (OSCC) are more detected after EBV infection. EBV produces a variety of proteins that are expressed at different times after B cells become infected (43, 44). These proteins consist of Epstein–Barr nuclear antigens (EBNA-2, EBNA-1, EBNAs-3A, 3C, 3B, and EBNA-LP), a viral counterpart to BamHI-H, BCL-2 rightward open reading frame 1, and latency membrane proteins 2 and 1 (LMP-2 and LMP-1) (44). Research has been conducted on these EBV products to explore their possible involvement in cancer development by supporting key characteristics of tumors (44). EBNA-1 is the main protein that keeps the virus in a dormant state before it begins to replicate and it controls various cellular genes by attaching to super-enhancer areas in the cell’s chromatin, which finally triggers the cell cycle to promote growth. LMP-1 is the primary oncoprotein among the latency-associated proteins and is frequently found in cancers associated with EBV (43). It engages with molecules that communicate signals from tumor necrosis factor (TNF) receptors, leading to the stimulation of various signaling pathways such as JNK–p-38, NF-κB, PI3K–AKT, ERK–MAPK, and JAK–STAT (44, 45). Numerous studies have indicated that activating these signal transduction paths promotes a wide range of downstream effects, including the regulation of adhesion protein expression. Additionally, it has been demonstrated to increase telomerase activity through the initiation of cMyc, while also facilitating the movement of tumor cells by triggering the stimulation and release of various matrix metalloproteinases. In addition, the protein has been observed to promote epithelial-mesenchymal transition in nasopharyngeal carcinoma (NPC) and exhibit characteristics similar to cancer stem cells. Besides LMP-1, there are also some other proteins which can be encoded by EBV and take part in its carcinogen activities (Figure 3) (42, 44).

#### 2.2.5 Other risk factors

Areca Nut (Betel Quid) is one of the potential risk factors for HNSCC which is mostly observed to be consumed in Southeast and South Asia and Polynesia, Solomon Islands, and Sri Lanka (46, 47). The areca nut which known as betel nut, is the fruit produced by the areca palm (*Areca catechu*). Areca nut chewing involves the consumption of areca nuts to experience their stimulant and narcotic effects (48). Approximately 600 million people globally consume areca nuts, which ranks as the fifth most widely used psychoactive substance after alcohol, caffeine, nicotine, and cannabis. In certain countries, areca nuts serve as the cheapest and most readily available stimulant and appetite suppressant, contributing to their widespread use among rural and low-income communities (49). A meta-analysis in this field has confirmed that the risk of oral cancer rises with both the length of time and the frequency of betel quid chewing, reinforcing the evidence for a causal link. Their findings also revealed a positive relationship between exposure and risk, with higher frequencies and durations of use associated with greater risk. In Taiwan, China, the risk increased in a linear fashion, while in India, the increase in risk was slightly less pronounced at higher levels of exposure (49). Another risk factor is oral hygiene and microbiome. Changes in the oral microbiome have been recommended to play a function in the progress of cancers of oral cavity in 7 to 15% cases that cannot be accounted for by established risk factors (50). The importance of the oral microbiome in a clinical context is highlighted by the statistical link between dysbiosis, often resulting from inadequate oral hygiene, and the occurrence of various cancer types. Within the diverse environment of the human mouth, which features mucosal areas and deep tissue gaps, both healthy and cancerous regions house unique microbial communities (51).

Age and gender are two other risk factors which put individuals at the risk of HNCs. According to an investigation conducted in 2012, there were 300,000 subjects of oral and lip cancers, representing 2.1% of the global total, with about two-thirds of the cases in men (52). Melanesia had the highest incidence rates for both genders, with 22.9 per 100,000 for males and 16.0 per 100,000 for females. Additionally, men in South-Central Asia and Eastern and Central Europe also experienced relatively high rates. According to this study, pharyngeal cancer also occurred significantly more regularly in males compared with females, with a sex ratio of 4:1. For both genders, the area with the highest incidence was Western Europe, which had rates of 7.5 per 100,000 for males and 1.6 per 100,000 for females (52). Laryngeal cancer also occurs particularly often in men, making up 1.9% of all cancer subjects in this group. The male-to-female ratio is 7:1, which is higher than for cancers in other locations. In women, it is relatively uncommon, with only about 19,000 new cases projected for 2012. Among men, high-risk areas include the Southern Europe (7.2), Caribbean, Eastern Europe and Central (7.9 cases per 100,000), and Western Asia (6.5) (52). In age point of view, Age plays an important function in the prognosis and incidence of HNCs, with the majority of cases occurring in older adults. The risk of increasing these cancers rises with age, often due to cumulative exposure to the mentioned risk factors (53). Additionally, age-related changes in immune function may affect tumor development and response to treatment. Older patients may also present with more progressive disease at diagnosis and can have comorbidities that complicate treatment options and outcomes. Understanding the impact of age on HNCs is crucial for tailoring prevention, screening, and treatment strategies for different age groups (53).

Exposure to industrial chemicals in the workplace has also been linked to negative health effects including HNCs for a long time, with the most significant levels of exposure found in the construction and furniture sectors and leather industries. The relationship between exposure to HNSCC and wood dust has been slightly debated in the literature and several studies have looked into this connection, especially regarding laryngeal and hypopharyngeal cancers, but fewer have focused on oral cavity and oropharyngeal cancers, yielding mixed findings. Furthermore, this relation is similar between leather dust and laryngeal cancer while pharyngeal cancer is confirmed to be associated with leather dust. On the other hand, studies have also recognized exposure to metal dust in the workplace as a risk-factor laryngeal cancer and a higher risk among individuals employed in metalworking jobs (54).

### 2.3 HNC treatment

**Figure 4:**
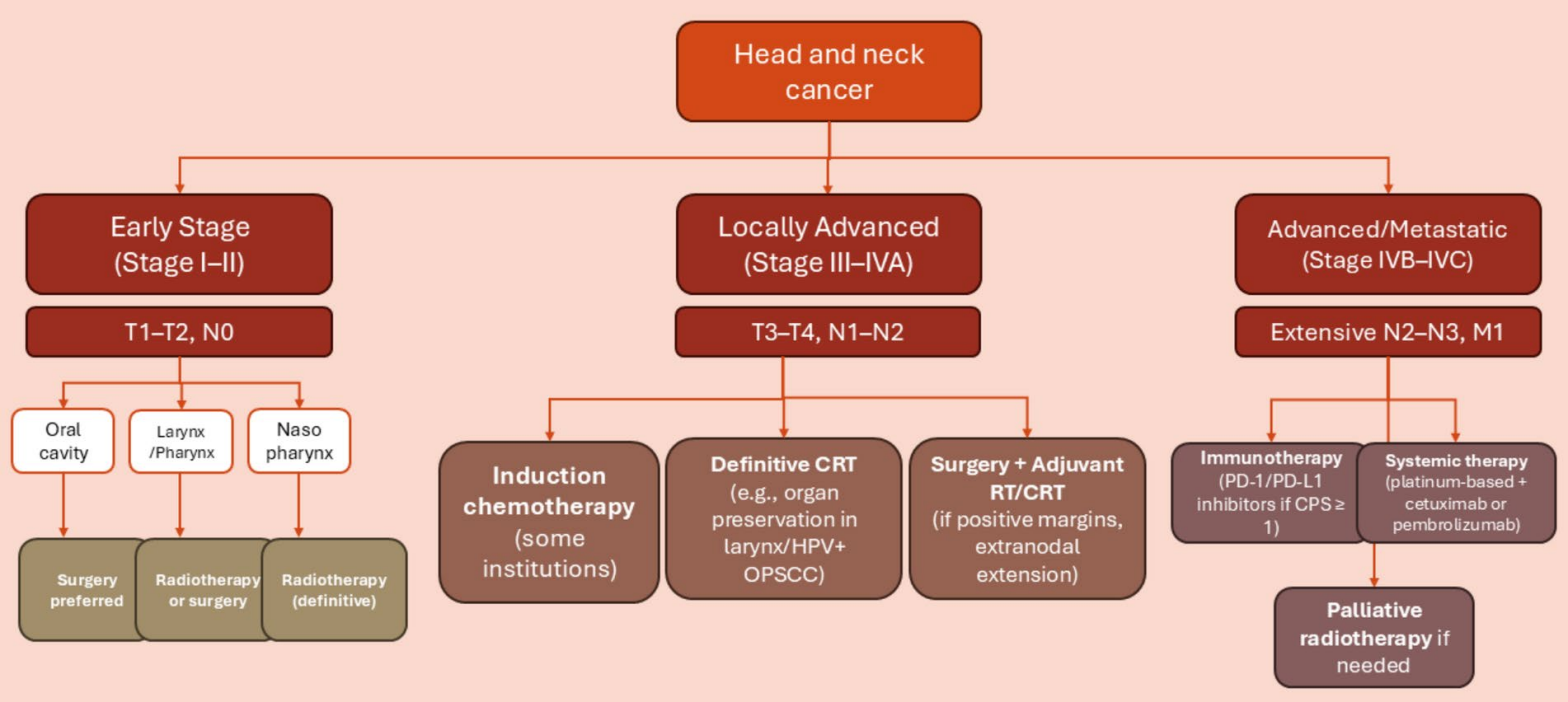
Decision tree outlining typical treatment pathways for head and neck squamous cell carcinoma (HNSCC) based on tumor location and TNM stage. Early-stage disease (Stage I–II) is often treated with a single modality such as surgery or radiotherapy, depending on the primary site. Locally advanced cases (Stage III–IVA) typically require multimodal treatment, including surgery followed by adjuvant chemoradiotherapy or definitive chemoradiotherapy. Advanced or metastatic disease (Stage IVB–IVC) is primarily managed with systemic therapy, including platinum-based chemotherapy and/or immunotherapy. Tumor location (e.g., HPV-positive oropharynx) and patient-specific factors (e.g., performance status, organ preservation goals) influence treatment selection.

The treatment of HNSCC involves using surgery, radiation, and chemotherapy in different combinations, depending on the TNM (node, tumor, and metastasis) stage and the primary location of the cancer (also summarized in Figure 4).

#### 2.3.1 Surgery

The primary treatment HNSCC involves surgery for tumors that can be surgically removed with clear margins. Depending on specific characteristics and the tumor’s anatomy, traditional open surgery or less invasive methods like laser surgery or transoral robotic surgery (TORS) are used for resecting the tumor (55). At present, TORS is available as an alternate option to chemoradiation, serving as a function-preserving method with or without neck dissection (ND). When performed by skilled professionals, TORS seems to be both effective and safe from an oncological perspective for specific cases of HNSCCs (55). Traditional open surgeries, which frequently involve procedures like lip splitting, lateral pharyngotomy, mandible splitting, or tongue resection through floor of mouth release, can lead to important collateral damage, resulting in cosmetic and functional impairments. Consequently, these surgical options are not very appealing. For subjects with HPV-driven oropharyngeal squamous cell carcinoma (OPSCC), TORS may offer a primary treatment that is equally effective and oncologically safe, while also associated with lower morbidity (55).

#### 2.3.2 Radiotherapy

Radiation therapy (RT) is another common treatment for HNSCCs. In the managing of locally advanced disease, RT is used as a supplementary treatment alongside surgery or in combination with chemotherapy. Also, the radiation dose for HNSCC typically ranges from 60 Gy to 70 Gy, depending on whether the treatment is adjuvant or definitive (56). In a definitive context, the Radiation Therapy Oncology Group (RTOG) 0129 phase 3 trial assessed both standard and accelerated boost radiation protocols with concurrent cisplatin, revealing no significant differences in regional or local control, overall survival, or late side effects (57). The likelihood of long-term toxicity from radiation rises when doses exceed 55 Gy to the pharyngeal constrictor muscles, salivary glands, and thyroid gland, potentially resulting in dysphagia, xerostomia, reliance on percutaneous endoscopic gastrostomy tubes, hypothyroidism and chronic aspiration. Recent advancements in intensity-modulated radiotherapy have made it possible to create conformal fields and apply dose constraints to the treated volume of the salivary glands (58). There are various ways to lower toxicity, one being the reduction of the conventional dose of definitive radiotherapy (70 Gy). Patients who tested positive for HPV underwent induction chemotherapy (ICT) and then established a lower-dose chemoradiation if they had a positive response to ICT (55).

#### 2.3.3 Chemotherapy

Chemotherapy has been utilized as part of the initial curative multimodal approach in the context of IC, alongside radiation therapy, and as a follow-up treatment. High-dose cisplatin continues to be the primary radiosensitizer used in the treatment of HNSCC. A meta-analysis indicated an absolute benefit from chemoradiation of 7% at 2 years and 8% at 5 years (59). Comparing radiation alone, induction chemotherapy with 5-fluorouracil and cisplatin followed by radiation, and concurrent administration of radiation and cisplatin for organ preservation in subjects with stage III and IV locally advanced laryngeal cancer, definitely excluding bulky T4 and T1 tumors indicates that long-term follow-up results showed a notable enhancement in larynx preservation rates and local control at both 5 and 10 years for the group receiving concurrent radiation and cisplatin compared with those undergoing induction therapy or radiation alone, with no significant differences in overall survival among the three treatment options. Consequently, the combination of radiotherapy and cisplatin is regarded as the standard care in North America (60).

#### 2.3.4 Immunotherapy

Recent investigations have shown that chemotherapy can be replaced by immunotherapy in HNC treatment. EGFR monoclonal antibodies cetuximab and panitumumab are the most investigated immunotherapies. In a trial by Bonner et al. (61), which focused on locally advanced HNSCC, researchers compared radiation therapy alone to radiation combined with weekly cetuximab. The data showed a substantial improvement in overall survival (29.3 months for radiation alone versus 49 months for the combination) and a longer period of locoregional recurrence-free survival (14 months compared with 24.4 months) with the combined treatment (61). Long-term follow-up over 5 years indicated that the benefits were mainly observed in patients with OPSCC, those under 65 years of age, and individuals who received a concomitant boost of radiation instead of standard once-daily radiation (61). Similar toxic effects were detected when panitumumab was combined with radiotherapy and cisplatin, resulting in poorer survival compared with just radiotherapy and cisplatin (62). Other EGFR inhibitors which can be used for HNCs treatment are Zalutumumab, Nimotuzumab, ABT-806, and MEHD7945A. Furthermore, besides EGFR inhibitor, other monoclonal Abs include PD-L1 inhibitors, PD-1 inhibitors, HGF inhibitors, VEGFR inhibitors, and CD antigen inhibitors (63). In summary, the incidence and mortality rates of HNSCC are changing globally, influenced by an aging population and shifts in cancer risk factors linked to socioeconomic development. Over the past decade, these cancers have increased especially among younger women in developed countries due to changing tobacco and alcohol use patterns and HPV prevalence. The growing number of HNSCC cases has attracted the attention of scientific society for finding more ways for overcoming these cancers. In the next section, we would discuss how exercise can be used for cancer treatment and further, we would investigate the role of exercise in HNCs.

## 3 Exercise and cancer

Exercise can be used besides the standard treatment of each type of cancer for many purposes. For instance, after receiving a cancer diagnosis and before initiating the treatment, patients typically face a normal waiting period of over 1 month before undergoing surgery, during which they often experience heightened levels of anxiety, depression, and a sense of loss (64). Furthermore, individuals who are in poorer physical and psychological condition prior to surgery are not only likely to have a delayed recovery post-operation (resulting in higher risk of complications and longer hospital stays) but also face an augmented risk of overall mortality after surviving cancer (65). Consequently, the time frame between the start of treatment and diagnosis has been identified as further than just a waiting during; it is seen as a crucial chance to make subjects for the demanding nature of cancer treatments (64, 65). Furthermore, in this time, exercise can also be an aid for cancer prehabilitation. Cancer prehabilitation is a focused approach that occurs between a cancer diagnosis and the start of intensive treatment, aimed at reducing the physical and emotional challenges associated with cancer therapy. Exercise plays a vital role in this prehabilitation process, positively impacting the subjects treatment outcomes and recovery after surgery (66, 67).

On the other hand, exercise can also be used for increasing the quality of cancer patients’ lives after receiving treatment. Also, surgery is one of the key methods for curing cancer or performing partial or complete tumor removal which despite improvements, around one-third of cancer people who have surgery face different postoperative problems and complications, including muscle loss, pain, and reduced physical function (68). Postoperative exercise, often referred to as rehabilitation, plays a significant role in aiding patient recovery and improving outcomes. Early mobilization, as a key aspect of postoperative exercise, can effectively lower the risk of cardiopulmonary complications, prevent blood clots, relieve pain, reduce the chances of bowel obstruction, and boost gastrointestinal function (69, 70). Post-surgery exercise for breast cancer subjects reduced pain, enhanced shoulder function, and increased QoL, aiding their recovery following surgery (71). A meta-analysis involving patients with lung and colorectal cancer indicated that exercising after surgery boosts health-related QoL, improves physical capabilities, and helps in managing fatigue (72). Moreover, it has been noted that exercise has beneficial effects during non-surgical treatments such as radiation therapy, chemotherapy, targeted therapy, hormone therapy, and immunotherapy. Most of the investigations in this area, which are conducted on breast and colorectal cancer, are showing that exercise notably decreases fatigue throughout adjuvant therapies, such as chemotherapy and/or radiation (73). As well, the results of these studies also indicated that implementing exercise throughout adjuvant therapy notably enhanced muscular strength, cardiorespiratory fitness, and lean mass among breast cancer subjects (74, 75). A study highlighted further advantages related to breast cancer-specific issues, including upper body and shoulder mobility, arm movements, and lymphedema through treatment, showcasing the effectiveness of exercise as a rehabilitation method following surgery. However, for colorectal cancer subjects, the mentioned effects are not reported (74). An important factor of physical activity during non-surgical cancer therapies is its role in enhancing treatment-related results, including how well patients can tolerate treatment, changes in cancer-related biomarkers, and the likelihood of cancer returning. Nevertheless, there is still a lack of adequate evidence on these outcomes (76).

## 4 Exercise and head and neck cancers (HNCs)

There is a limited number of studies probing the impacts of exercise on different HNCs; however, we have gathered as many evidence as we could and we have also summarized them in Tables 1–3.

**Table 1:** A summary of investigations examining exercise on head and neck cancer patients focusing on reducing trismus.

Type of cancer	Participants	Intervention	Duration	Results	Reference
Head and neck cancer	27 patients	TheraBite^®^ Jaw Motion Rehabilitation System™ vs. Dynasplint Trismus System^®^.	3 months	No notable differences between devices, MMO increase of 3.0 mm with the first tool and 1.5 mm with the second tool.	(77)
Oral and/or oropharyngeal tumors	100 patients	5–6 times for 45 min in a week	12 months	Early supervised exercises combined with self-care treatment do not have additional benefits compared with usual care during curative radiotherapy	(80)
	37 patients	Early vs. late jaw exercise	6 months	The timing of the exercise did not affect the outcomes and a wider jaw opening linked to enhanced quality of life	(78)
Oral cancer	60 subjects (30 intervention and 30 control)	Warm compress, masticatory muscle massage, and jaw exercise	12 weeks	Change in maximum interincisal opening was 10.30 mm (95% CI: 8.22–12.37) The change in mandibular function impairment score was −0.36 (95% CI: −0.44 to −0.28)	(79)

**Table 2:** Studies investigating the role of exercise on dysphagia in HNC patients.

Type of cancer	Participants	Intervention	Duration	Results	Reference
Laryngeal cancer	6 participants who had previously undergone SCPL	Expiratory muscle strength training (EMST)	4 weeks	This method only improved Peak cough flow (PCF)	(82)
	78 patients with total laryngotomy	7 flexibility exercises for the head, neck 8 range-of-motion exercises for the tongue, lips, and jaw, and, 5 lymphedema exercises.	12 weeks	The progression of overall swallowing difficulties was decreased in the intervention group (*p*-value for two-way interaction = 0.013)	(84)
	92 patients undergoing total laryngectomy	Weekly swallowing therapy	12 months	The scores were significantly better among the intervention patients at months 3, 6, 9, and 12 for all the scores, with *p* values smaller than 0.000	(85)

**Table 3:** Studies investigating the role of exercise in vocal function in HNC patients.

Type of cancer	Participants	Intervention	Duration	Results	Reference
Laryngeal cancer	10 adults irradiating for laryngeal cancer	Vocal function exercises	6 weeks		(88)
	11 patients who underwent SCLC with cricohyoidoepiglottopexy (CHEP)	Vocal exercise involving prolonged /b/ sound and the vowel /e/ with chest and arm-pushing exercises	5 series of exercises	Improving the general level of vocal, roughness, and breathiness after the fourth series of exercises	(83)
	78 patients with total laryngotomy	7 flexibility exercises for the head, neck 8 range-of-motion exercises for the tongue, lips, and jaw,	12 weeks	No significant effect on the speech issues	(84)

To support safe and effective exercise prescription across treatment phases, we referenced the American College of Sports Medicine Guidelines for Cancer Survivors, which recommend tailoring intensity and modality based on treatment-related side effects, functional status, and comorbidities. For instance, low- to moderate-intensity resistance training (e.g., 30–60% of 1RM) is considered appropriate during radiotherapy when mucositis or fatigue are present, while aerobic training can begin at 40–60% of VO₂max and gradually progress. Several studies in HNC populations have reported a decrease in VO₂max by up to 30% during chemoradiotherapy, reflecting significant deconditioning. However, clinical trials show that structured rehabilitation post-treatment can significantly improve cardiorespiratory fitness, with VO₂max increases of 10–20%, even in previously sedentary HNC survivors. These findings highlight the importance of evidence-based, phase-specific exercise plans for improving outcomes in this population.

### 4.1 Trismus prevention with exercise

Trismus is a condition where individuals experience limited movement in the jaw muscles, making it difficult to open their mouths wider than 35 mm. This complication is observed after conventional treatments for HNCs including surgery. The current literature indicates that rehabilitation strategies designed to prevent trismus include both passive and active stretching exercises for the jaw, and may utilize jaw mobilization tools (all studies are summarized in Table 1). For instance, a group of researchers tried two different tools on HNC patients (77). Their study aimed to evaluate the efficiency of two extending devices for improving maximal mouth opening (MMO). A target sample of 30 patients per group was set and during the initial visit, patients received a stretching device. Results showed no noteworthy difference in MMO improvement between the two devices, with gains of 3.0 mm for TheraBite users and 1.5 mm for DTS users. Only one patient achieved full recovery from trismus (77). They also reported that patients found it difficult to use either stretching device because of the demanding exercise regimen, pain experienced during workouts, issues with properly using the stretching equipment, and their overall declining health (77).

Sandler et al. (78) examined Jaw variety of motion exercise therapy on patients with oropharyngeal and/or oral tumors who were scheduled to undergo surgery and did not use any tools in their study. They measured jaw ROM, pain numeric rating scales (NRS), patient-rated QoL and performance status scale-head and neck (PSS-HN) in their subjects and found out that the degree of jaw opening was significantly linked to several QoL assessments, such as the ability to eat normally and the comfort of eating in public. Moreover, it was seen the relationship between MDADI global and jaw MIO was stronger 1 month after the operation compared to 2–3 months or 6 months’ post-surgery. However, this relationship weakens in the months after the surgery (78). However, this study did not assess the start of exercise at 1, 2–3, and 6 months after surgery. Although the changes between the results of the two exercise intervention protocols were not statistically significant, this study provides a valuable basis for future research into the potential advantages of starting jaw exercises early versus later. The preliminary results, along with the nearly significant correlation between maximum interincisal opening (MIO) and MDADI Global scores. However, the small sample size and other limitations highlight the need for a follow-up study with a larger participant group, an extended time frame between interventions, and a well-defined control group to better assess the ideal timing and effectiveness of postoperative jaw exercise interventions (78).

Another study on oral cancer patients also confirms the impact of exercise in decreasing trismus and improving mandibular function in subjects undergoing curative surgery for oral cancer (79). In study, people who were admitted for oral cancer surgery at a general hospital were recruited and divided into either the active control group or the experimental group. Both groups participated in a 12-week intervention program that included warm compresses, massages for the masticatory muscles, and jaw exercises (79). These subjects were assessed at the beginning of the study, at week 4, and week 12. The results indicated that after 12 weeks of intervention, the experimental group engaged in 299.67 min more practice time than the active control group. The increase in maximum interincisal opening was 10.30 mm higher in the experimental group compared with the active control group. Additionally, the change in the mandibular function impairment score was −0.36 more favorable in the experimental group than in the active control group (79). Despite the promising findings of the mentioned studies, another investigation showed paradoxical results. In a study involving subjects with HNCs receiving radiotherapy, participants were assigned to either a group that engaged in supervised exercises 5–6 times a week for 45 min, in addition to standard care, or a control group that received only standard care. The main goal was to assess changes in maximal interincisor distance (MID) at 5 and 12 months, while secondary outcomes included cervical range of motion, tissue tightness, and QoL (80). At 12 months’ post-radiotherapy, the mean difference in MID for the exercise group compared to the control group was 0.83 mm, indicating no significant improvement. After adjusting for surgery and tumor size, the adjusted mean difference was 5.92 mm, still not statistically significant (80). Notably, cervical rotation significantly worsened in the exercise group compared to controls, while other secondary outcomes showed no significant changes. They concluded that early supervised exercises do not appear to offer additional benefits over standard care in reducing trismus for patients with oral cavity or oropharyngeal cancer during radiotherapy (80). In summary, current research evaluates rehabilitation strategies, including the use of devices like TheraBite^®^ and Dynasplint Trismus System^®^, to improve maximal mouth opening (MMO). One study found no noteworthy change in MMO improvement between the devices, with minimal gains reported and challenges faced by participants. Another study examined jaw range of motion exercises without tools for patients undergoing surgery for oral and oropharyngeal tumors. It highlighted a correlation between jaw mobility and QoL measures, suggesting that early exercise initiation post-surgery could enhance QoL. However, the small sample sizes and other limitations designate the requirement for further research to better understand the impact of postoperative jaw exercises.

### 4.2 Dysphagia and swallowing exercises

Dysphagia, or difficulty swallowing, is a common and often distressing complication faced by subjects who have experienced treatment for HNCs. This condition can arise as a result of numerous treatment modalities, including radiation therapy, surgery, and chemotherapy, which can lead to functional and structural changes in the pharynx, oral cavity, and esophagus. After surgical intervention, patients may experience alterations in anatomy, such as the removal of tumors or surrounding tissues, which can impact the swallowing mechanism. Radiation therapy can cause inflammation and fibrosis in the tissues of the throat, leading to decreased mobility and sensitivity in the swallowing structures. Chemotherapy may further exacerbate these issues by causing mucositis, which can make swallowing painful and difficult. The consequences of dysphagia extend beyond the physical act of swallowing; they can significantly affect a patient’s nutritional status, hydration levels, and overall QoL. Given the importance of dysphagia, a great body of research has tried to overcome this side effects through a diversity of interventional methods. The most recent investigation in this field is conducted in 2024 which is a case report on a 72-year-old subject who was diagnosed with larynx carcinoma and underwent SCPL with cricohyoidoepiglottopexy (CHEP) (81). In the fiberoptic endoscopic evaluation of swallowing assessments, the patient consistently showed signs of laryngeal penetration and potential tracheal aspiration, which were corroborated by videofluoroscopic swallowing study results. Furthermore, a narrowing of the upper segment of the cervical esophagus was noted, attributed to a large bone spur in the front part of the C5 vertebra. This narrowing hindered the movement of the bolus, causing it to accumulate above the upper esophageal sphincter and subsequently enter the airway (81). This patient received rehabilitation for 3 months and the data exhibited that the buildup of the bolus above the cervical osteophyte of patient was cleared with several swallows, showing no signs of penetration or aspiration in him. This case report highlights the need for a coordinated assessment that includes a variety of specialists such as otorhinolaryngologists, speech therapists, and radiologists to investigate dysphagia in patients receiving conservative laryngeal surgeries, so that rehabilitation can be tailored to their individual needs (81).

Expiratory muscle strength training (EMST) is one of methods in which patients are instructed to exhale vigorously using a device equipped with a one-way spring-loaded valve. This adjustable valve prevents airflow until enough expiratory pressure is generated, at which point it opens to let the air flow through. The valve can be modified to accommodate different maximum expiratory pressure (MEP) levels, enabling the control of airflow resistance tailored to each individual, which can be progressively heightened as needed (82). Palmer et al. (82) used this method for six patients who had undergone supracricoid partial laryngectomy. Their results shown that the average MEP raised during follow-up, which was not statistically significant. Since EMST focuses on expiration rather than inspiration, an increase in maximum inspiratory pressure from baseline to follow-up was not anticipated, and this expectation was confirmed (82). Overall, patients were able to undergo a 4-week treatment program of EMST following SCPL with very few side effects. The main advantages seemed to center around breathing and coughing, rather than voice and swallowing. Nonetheless, these benefits are significant, considering how vital these functions are for respiratory health, especially in individuals who are prone to aspiration (82). Another similar study worked on patients who underwent supracricoid laryngectomy with CHEP and detected an enhancement in the overall level of deviations concerning vocal quality, roughness, and breathiness of the patients. They concluded that the extended vocal exercise involving the prolonged /b/ sound along with the vowel /e/, paired with chest and arm-pushing exercises, showed positive effects on the overall vocal quality, specifically in reducing roughness, deviation, and breathiness. These improvements began to be noticeable from the fourth minute in subjects who had experienced supracricoid laryngectomy with CHEP reconstruction (83).

Another study probed the condition of patients with total laryngotomy (TL) and tried to enhance their swallowing problems through a guided self-help exercise intervention which contained flexibility exercises for head, neck, jaw, tongue, and lips. They used the swallowing QoL questionnaire (SWAL-QoL) foe assessing the problems of patients and observed that the total swallowing problems improved significantly over time in the intervention group compared with the control group. The data revealed that, at the 6-month follow-up, subjects in the intervention group had a significantly lower score than those in the control group (84). There were seven subdomains in SWAL-QoL score including fear of eating, eating duration, food selection, general burden, mental health, eating desire, and social function. These subdomains did not exhibit a significant change over time separately; nevertheless, the intervention group had meaningfully improved (lower) scores at the 6-month follow-up for eating duration and fear of eating (84). Social Functioning and mental health had also notable differences which was significant (84). In prophylaxis point of view, research evaluated the efficiency of preventive swallowing exercises on the swallowing abilities of patients who were having a total laryngectomy (TL). They included 92 patients who had a TL due to stages III and IV laryngeal cancer to their study and practiced them for five specific swallowing exercises for the duration of 3 months following their surgery, beginning 2 weeks after the procedure. Weekly therapy sessions were also conducted with the patients. The results shown that there were no significant differences in the swallowing function, eating in public subscale, and normalcy of diet subscale scores between the intervention and control groups; at months 3, 6, 9, and 12, the scores for the intervention subjects were significantly improved across all measures (85).

A study compared a resistance-based chin-to-chest (CtC) exercise with a head-lift exercise for achieving better measures of hyolaryngeal muscle activation in healthy participants (86). A decrease in the opening of the upper esophageal sphincter (UES) during swallowing is a key factor in dysphagia, which can be caused by both neurological and cancer-related issues. The UES normally opens due to factors like relaxation controlled by the central nervous system, pressure from the bolus at the UES, and the movement of the hyoid and thyroid bones (hyolaryngeal excursion). This movement is driven by muscles like the mylohyoid and thyrohyoid. When hyolaryngeal excursion is reduced, the UES does not open enough, which can prevent the bolus from entering the esophagus and increase the risk of aspiration, potentially leading to serious health issues. If untreated with swallowing therapy, this may require feeding tubes for proper nutrition. Participants lifted and held their heads from a lying position for 10 s. The activation of the hyolaryngeal muscles, as assessed through surface electromyography (sEMG), was notably higher during the CtC exercise than the other exercise (86). However, this study was done on healthy individuals and further investigations are needed for using CtC on HNC patients.

### 4.3 Vocal exercises

Chronic vocal dysfunction is a common and often debilitating consequence of treating HNCs, which can involve surgery, radiation therapy, and chemotherapy. These treatments can lead to various vocal issues, including hoarseness, breathiness, reduced vocal range, and difficulty with projection. The underlying causes of these dysfunctions may include damage to the laryngeal structures, changes in vocal fold elasticity, and alterations in the surrounding musculature and nerves. Research indicates that the incidence of chronic vocal dysfunction may significantly affect patients’ QoL, impacting their ability to communicate effectively and engage socially. Voice therapy, which may include exercises to strengthen vocal muscles and improve technique, is often recommended as part of rehabilitation. Additionally, interventions such as surgical procedures or the use of voice prostheses may be considered for more severe cases. Ongoing studies aim to better understand the long-term impacts of cancer treatments on vocal function and to develop effective strategies for prevention and rehabilitation. Early intervention and tailored therapeutic approaches are crucial for improving outcomes and elevating the overall QoL for survivors of HNCs experiencing chronic vocal dysfunction (87).

In this point of view, there are plenty of studies which have focused on solving this problem by vocal exercises. Angadi et al. (88) tried to increase the voice-related QoL of laryngeal cancer patients and used a combination of vocal hygiene counseling (VH) and vocal function exercises (VFEs). Their research showed that both groups demonstrated an overall enhancement in acoustic parameters. The VFE + VH group showed significant improvements in maximum phonation time and pitch range. However, the VH group did not show significance in any of the mentioned parameters. In contrast, the VH group did not exhibit any changes in these stroboscopic parameters from pre- to post-treatment. Additionally, high-speed imaging showed notable pre- to post-treatment changes in the amplitude of vibration for the VFE + VH group, while the VH group did not demonstrate any significant alterations in high-speed parameters (88). A multicenter study in Netherlands worked on HNC patients who had a total laryngotomy surgery (84). They used several exercises for these patients including eight range-of-motion exercises for the lips, tongue, and jaw which were taught by speech therapist to patients. In this study, after a 6-months follow up, no notable main effect was observed on the speech issues. Nevertheless, moderation analyses indicated positive impacts on the SHI for a small group of subjects within 6 months following TL surgery (84).

### 4.4 Other effects of exercise on HNC patients

TL combined with ND is a fundamental treatment for subjects with advanced-stage laryngeal cancer. While this surgical intervention can significantly improve survival rates, it is associated with various postoperative complications. One common and well-documented complication is shoulder dysfunction, which can negatively influence a patient’s QoL. This dysfunction typically results from the disruption of the spinal accessory nerve during the neck dissection procedure, leading to limitations in shoulder mobility and strength. Recognizing the impact of shoulder dysfunction on recovery, healthcare providers have begun exploring early rehabilitation strategies to mitigate these effects. Prophylactic rehabilitation, implemented shortly after surgery, aims to prevent or reduce the severity of shoulder dysfunction and enhance recovery. In this regard, a study on 76 people who underwent TL with ND tried to answer the questions about the impacts of exercise on shoulder dysfunction (89). The control group underwent the usual standard care without any structured shoulder exercises, whereas the intervention group participated in a preventive rehabilitation program that lasted 12 weeks. Participants were evaluated at the beginning of the study, as well as 3- and 6-months after surgery (89). The results indicated that there was a significant improvement in shoulder function and QoL reported by patients over time. However, there was no notable change between the intervention and control groups, which suggests that the preventative intervention had little to no impact on shoulder function outcomes (89). Another study also worked on shoulder problems in patients who underwent TL from 5 different HNC centers in the Netherlands (84). This research was consisting of two treatment groups: intervention and control. Patients in the intervention group received both a self-care education program and a guided self-help exercise program, while the control group only received the self-care education program (84). Strengthening exercises were excluded from this intervention despite their recognized significance in rehabilitation, as their focus was solely on incorporating simple exercises. Their results shown that there were no notable changes in shoulder issues over time in the intervention group compared with the control group. While statistically significant differences in shoulder problems were observed at the 3- and 6-months follow-up, these differences lost their statistical significance after adjusting for the baseline scores (84). Also some differences in health-related QoL (HRQoL) of intervention group at 3 and 6-months follow-up were detected but they were not statistically significant (84).

The safety and efficacy of exercise during different phases of HNC treatment depend on treatment intensity, symptom burden, and individual capacity. Prehabilitation, initiated after diagnosis but before treatment, is generally safe and improves post-surgical outcomes, physical function, and mental resilience. During active treatment, such as radiotherapy or chemotherapy, moderate-intensity aerobic or resistance exercise has been shown to mitigate fatigue, preserve muscle mass, and enhance quality of life (90, 91). However, high-intensity exercise (e.g., HIIT) should be used cautiously, particularly during radiotherapy, when mucosal inflammation, dysphagia, and fatigue are common. In such cases, aggressive exercise may exacerbate tissue injury, delay healing, or worsen symptoms. Thus, exercise interventions should be individualized, closely monitored, and preferably supervised by exercise physiologists or oncology rehabilitation specialists. Post-treatment exercise plays a crucial role in recovery and rehabilitation, aiding in the restoration of physical capacity, speech and swallowing function, and psychological well-being (90, 91).

## 5 The effects of combined polyphenol and exercise on brain function related to neurodegenerative diseases

### 5.1 Crocin

A recent study investigated the combined effects of AE and crocin supplementation on learning, memory, and hippocampal gene expression of neurotrophins and tau in an Alzheimer’s disease (AD)-like rat model. Forty male Sprague Dawley rats were assigned to five groups: healthy control, endurance training (ET), crocin treatment, Alzheimer’s control, and combined ET plus crocin. Alzheimer’s was induced in all groups except the healthy control via intraperitoneal injection of trimethyltin chloride (TMT). ET involved treadmill running three times a week, while crocin was administered daily at 25 mg/kg. TMT-induced Alzheimer’s significantly impaired memory and learning and downregulated NGF, TrkB, and BDNF gene expression, while upregulating tau gene expression. Both ET and crocin independently improved cognitive functions and neurotrophin gene expression while decreasing tau expression. Notably, the combination of ET and crocin produced the most substantial improvements, highlighting a synergistic effect on neuroprotection and cognitive function in the AD model (92) (Table 4). Another study investigated the combined effects of ET and crocin on aerobic power and anxiety-like behaviors in AD model rats. Forty rats were distributed into five groups: control, ET, ET plus crocin (ETCR), crocin, and sham. Over 8 weeks, rats in ET and ETCR groups performed treadmill running three times per week, while the ETCR and crocin groups also received daily crocin injections (25 mg/kg). The data showed that ET alone meaningfully reduced anxiety-like behaviors and body weight, and improved aerobic capacity. Crocin administration also significantly enhanced aerobic power and reduced anxiety-like behaviors. However, combining ET with crocin did not show additional synergistic benefits beyond the effects of each treatment alone (93).

**Table 4:** The effects of combined polyphenol and exercise on brain function related to neurodegenerative diseases.

Polyphenol	Participants	Diseases	Findings	Ref
Crocin	Rats	Alzheimer’s disease	Increased memory, learning, and neurotrophin gene expression and decreased tau gene expression	(92)
Crocin	Humans	Alzheimer’s disease	Training and crocin distinctly function and from different pathways effective in decreasing anxiety	(93)
Crocin	Rats	Alzheimer’s disease	Increased IGF-1 content of the hippocampus	(94)
Crocin	Rats	Alzheimer’s disease	Improved gene expression of calmodulin kinase 2	(95)
Quercetin	Rats	Alzheimer’s disease	Improved antioxidant defense system and STZ-induced memory impairment	(96)
Resveratrol	Rats	Alzheimer’s disease	Improved aortic morphology with no change in pulse wave velocity	(97)
Resveratrol	Rats	Alzheimer’s disease	Reduced toxicity of Aβ oligomers, suppression of neuronal autophagy, decreased apoptosis, and upregulation of key growth-related proteins	(98)
Resveratrol	Rats	Alzheimer’s disease	Improved fracture resistance and cross-sectional geometric indicators of bone strength	(99)
Resveratrol	Rats	Alzheimer’s disease	Improved hippocampal function	(100)
Resveratrol	Rats	Alzheimer’s disease	Increasing the expression of BDNF, VEGF, and FGF7 genes in the brain tissue	(101)
Grape juice	Rats	Parkinson’s disease	Reduced oxidative damage	(102)
Grape juice	Rats	Parkinson’s disease	Improved BDNF levels	(103)
Grape juice	Rats	Parkinson’s disease	Improved antioxidant agents	(104)
Grape juice	Rats	Parkinson’s disease	Improved dopamine concentrations	(105)
Grape juice	Human	Multiple sclerosis	Improved cognitive disorders (memory)	(106)
Grape juice	Human	Parkinson’s disease	Reducing oxidative damage and preserving cognitive function	(107)
Curcumin	Rats	Parkinson’s disease	Inhibiting oxidative stress indices and regulating behavioral tasks	(108)
Curcumin	Rats	Alzheimer’s disease	Improved Aβ levels in the hippocampus	(109)

A study explored the effects of ET combined with crocin intake on glycogen and IGF-1 expression in the hippocampus of rats with TMT-induced AD. Thirty rats were allocated into five groups: Alzheimer’s control, healthy control, ET, crocin, and ET with crocin. AD was induced in groups 2–5 using 8 mg/kg TMT. Over 8 weeks, rats in the training groups ran on a treadmill three times per week, and crocin-treated groups received daily 25 mg/kg crocin. The results showed that TMT significantly reduced glycogen and IGF-1 gene. ET alone significantly increased IGF-1 and glycogen, while crocin alone had no significant effect. However, combining ET with crocin led to significant improvements in IGF-1 expression and glycogen levels, suggesting that endurance exercise, with or without crocin, plays a key role in counteracting TMT-induced deficits (94). Another study investigated the effects of ET combined with saffron (Sa) intake on calmodulin kinase 2 and cytochrome C expression in the heart tissue of AD model rats. Forty rats with TMT-induced AD were allocated into five groups: ET, AD control, Sa, ET + Sa, and sham. Over 8 weeks, rats in the ET and ET + Sa groups performed treadmill running (15–20 m/min, three sessions per week), and the Sa and ET + Sa groups received 25 mg/kg of Sa extract daily. The findings showed that Sa, ET, and ET + Sa treatments each significantly decreased calmodulin kinase 2 and cytochrome C levels. Moreover, the combination of ET and Sa led to a greater reduction in calmodulin kinase 2 compared to ET alone (95).

It should be noted that the referenced crocin studies were conducted in male rats to reduce variability linked to hormonal cycles, which can affect behavioral and biochemical outcomes. However, future research should incorporate both sexes to fully elucidate potential sex-specific effects of crocin treatment.

### 5.2 Quercetin

A study investigated the synergistic influences of quercetin and exercise on memory and learning impairments in a streptozotocin (STZ)-induced AD rat model. Fifty-six Wistar rats were distributed into eight groups, including sham, control, AD, quercetin-treated, quercetin vehicle, exercise pretreatment, off-treadmill, and exercise + quercetin groups. Quercetin (80 mg/kg) was consumed intraperitoneally for 21 days post-STZ injection, while exercise involved treadmill running (1 h/day, 20–22 m/min) for 60 days. STZ administration led to spatial memory deficits and elevated hippocampal oxidative stress. Both exercise pretreatment and quercetin treatment individually improved memory and oxidative stress. Notably, their combination produced the most significant improvements, suggesting a synergistic effect on enhancing antioxidant defense and cognitive function (96).

### 5.3 Resveratrol (RES)

A study assessed cardiovascular function in 7-month-old male 3xTg AD mice and age-matched wild-type (WT) mice. While interventricular septal dimensions and aortic root were similar, 3xTg mice showed reduced systolic function and impaired diastolic function compared with WT mice. RES supplementation and treadmill exercise training for 5 months normalized diastolic deceleration time and improved systolic function. Although pulse wave velocity was ~33% higher and elastin fragmentation greater in 3xTg mice, exercise and RES improved aortic structure without significantly altering pulse wave velocity (97). Another study evaluated the effects of 5 months of ET, RES treatment, or their combination on Aβ toxic species and markers of apoptosis, inflammation, neuroprotection, and endolysosomal degradation in 3xTg-AD mice. RES treatment significantly reduced neuroinflammation and Aβ oligomer accumulation, enhanced neurotrophin and synaptic marker levels, increased silent information regulator expression, and decreased markers of apoptosis, autophagy, endolysosomal degradation, and ubiquitination. Exercise training alone improved some neuroprotective markers, but combining ET with Resv did not provide additional benefits beyond those achieved by Resv alone (98).

A study investigated whether RES and exercise could improve bone fracture resistance in a 3xTg-AD mouse model. 3-month-old male 3xTg-AD mice were treated for 4 months with RES, exercise, or both, and compared with WT mice. Exercise involved treadmill running (15 m/min, 45 min/day, 5 days/week), while RES was provided at 4 g/kg diet. The data revealed that 3xTg mice had weaker bone quality compared with WT mice. However, combined RES and exercise treatment meaningfully improved bone strength and quality, restoring them to WT levels (99). Another work examined the impacts of RES and aerobic training (AT) on the AMPK/PGC-1α/SIRT1 pathway in the hippocampus of AD rats. Wistar rats were distributed into 5 groups: Normal, AD, AD with training (ADT), AD with RES (ADRES), and AD with training plus RES (ADTRES). The RES groups received 20 mg/kg orally, and the exercise program involved treadmill running 5 days per week for 8 weeks. The data showed that AD meaningfully reduced AMPK/PGC-1α/SIRT1 expression, while both RSV and exercise treatment increased their expression. The combination of exercise and RES (ADTRES group) led to greater increases than either treatment alone (100).

A study explored the influences of AT and combined RES and fisetin (Fis) treatment on brain neurogenesis signaling pathways in AD mice. Twenty-five mice were divided into five groups: AD, AD+RES + Fis, control, AD + AT, and AD+AT+RES + Fis. AD was induced by injecting amyloid-beta into the hippocampus. AT was performed 5 days/week for 8 weeks, and RES (25 mg/kg) plus Fis (20 mg/kg) were administered. Results showed that AD reduced hippocampal BDNF, VEGF, and FGF7 gene expression, while AT and RES + Fis treatments significantly increased these neurogenesis-related markers (101).

### 5.4 Grape juice (GJ)

A study assessed the effects of GJ consumption combined with aquatic exercise on cognitive function and oxidative stress in people with Parkinson’s disease (PD). Participants were allocated into a grape juice group (GJG) and a control group, both undergoing 4 weeks of aquatic exercise. The GJG additionally consumed 400 mL of GJ daily. No cognitive improvements were seen in either group. Both groups showed reductions in TBARS, antioxidant enzymes, and uric acid, but only the GJG had a significant reduction in protein oxidation levels (102). Another work evaluated the effects of aquatic exercise combined with GJ consumption on BDNF, functional outcomes, and histone H4 acetylation in people with PD. Nineteen participants underwent 4 weeks of aquatic training, with one group also consuming GJ daily. Aquatic exercise improved mobility, functional capacity, BDNF levels, and histone H4 acetylation, but GJ did not further enhance these effects, as no significant differences were found between the groups (103).

The study assessed the effects of red GJ combined with treadmill running on a PD animal model using 30 male Wistar rats. The rats were allocated into five groups: PD, Sham, PD treated with exercise (PD-Ex), PD treated with GJ (PD-GJ), and PD treated with both GJ and exercise (PD-GJ-Ex), with six rats per group. The data showed a significant change in the number of rotations between the Sham and PD groups. At the end of the experiment, the number of rotations reduced meaningfully in both the PD-GJ-Ex and PD-GJ groups. Exercise alone did not significantly affect the number of rotations (104). Another study compared the neuroprotective effects of red GJ and exercise on PD in male rats. Two weeks after surgery, the rats were treated with red GJ and exercise for 1 month. The extent of the lesion was assessed using intraperitoneal apomorphine injections, and the number of rotations within 1 h after injection was used as the main evaluation parameter for PD. The results exhibited that red GJ meaningfully reduced the signs of PD compared with other groups. Rats with PD that did not receive red GJ had the highest number of rotations. Both red GJ and exercise reduced rotations, but red GJ was more effective in reducing PD symptoms than exercise alone (105).

A work examined the impacts of combined training and red GJ intake on memory improvement in women with multiple sclerosis. Forty-eight women aged 20–40 were distributed into four groups: supplement, training, training + supplement, and control. The combined training, consisting of 60-min sessions 3 times per week for 8 weeks, was performed at an intensity of 10–12 on the Rate of Perceived Exertion scale. Red GJ supplementation was also administrated 3 times/week for 8 weeks. Memory was assessed before and after the intervention using daily, prospective, and retrospective memory questionnaires. The findings exhibited significant improvements in daily, retrospective, and prospective memory in the training, supplement, and training + supplement groups compared with the control group (106). Another work aimed to compare the influences of different long-term exercise modalities on cognitive performance and antioxidants in PD patients. Sixty-one participants were allocated to either the AE, Tai Chi exercise (TCE), or control group. The results showed no significant changes in cognitive performance or oxidative markers in the TCE and AE groups. However, after 12 weeks of AE, there was a significant increase in catalase and GSH levels, alongside a decrease in uric acid (UA) levels. The TCE group showed a substantial rise in GSH levels. In contrast, the control group experienced a significant decrease in superoxide dismutase activity and Mini-Mental State Examination (MMSE) scores. Associations between changes in MMSE and changes in UA and GSH levels were significant in the AE group. The findings proposed that long-term AE and TCE may help preserve cognitive function and reduce oxidative damage in PD, with AE providing greater benefits than TCE (107).

### 5.5 Curcumin

A study explored the neuroprotective influences of curcumin and regular AE in PD. Rats were treated for 8 weeks with curcumin (50 mL/kg) alone or in combination with AE. PD was induced by 6-OHDA, resulting in increased α-synuclein protein levels, higher malondialdehyde levels, decreased substantia nigra neurons, total antioxidant capacity, and glutathione peroxidase activity in brain tissue. These alterations were reversed by the combined treatment of curcumin and AE. Behavioral tests confirmed these results, showing increased rod test time and rotations due to apomorphine injection. Histopathological assays supported the antioxidant and behavioral improvements observed (108). Another study investigated the effect of moderate-intensity interval training combined with curcumin consumption on LRP1 (a key carrier of Aβ) and Aβ levels in the plasma and brain of AD induced rats. Fifty Wistar rats were allocated into 5 groups: AD + exercise, AD + exercise + curcumin, AD, AD + curcumin, and control. After a 3-day recovery, the training groups underwent moderate-intensity interval training for 4 weeks, while the other groups continued their normal routine. Curcumin was injected three times a week into the curcumin groups. The results showed significant differences in hippocampal Aβ, plasma Aβ, and plasma LRP1 levels between the groups. The AD + exercise + curcumin group showed the highest improvements in plasma Aβ and LRP1 levels compared to both the healthy control and AD control groups (109).

## 6 Conclusion and future directions

Current evidence underscores the powerful interplay between polyphenol intake and exercise in regulating molecular pathways crucial for brain function, tumor suppression, metabolic homeostasis, and healthy lifespan extension. Both interventions individually promote antioxidant defenses, modulate inflammatory responses, enhance autophagy and apoptosis regulation, and support neuroplasticity and metabolic balance. When combined, polyphenols and exercise exhibit a synergistic effect, amplifying protective mechanisms against neurodegeneration, head and neck cancers, and age-related metabolic dysfunctions. However, despite promising preclinical and clinical findings, significant gaps remain. Future research should prioritize large-scale, longitudinal human studies to better define optimal types, dosages, and timing of polyphenol supplementation in conjunction with specific exercise regimens. Advanced multi-omics approaches, including epigenomics, metabolomics, and proteomics, will be critical for unraveling the precise molecular networks involved in this crosstalk. Furthermore, personalized strategies based on individual genetic, epigenetic, and microbiome profiles could enhance the efficacy of combined polyphenol and exercise interventions. In conclusion, integrating polyphenol-rich diets with regular physical activity holds great promise as an accessible, non-invasive, and cost-effective approach to improve brain health, suppress tumorigenesis, mitigate metabolic disorders, and extend the healthy human lifespan. Strategic interdisciplinary efforts will be essential to translate these insights into personalized, clinically applicable programs that promote resilience against cancer and neurodegenerative diseases across the aging spectrum.

Although numerous studies suggest a synergistic interaction between polyphenol intake and physical exercise, few quantify this synergy through formal statistical methods such as effect size calculations or interaction models. The term “synergy” is often used descriptively rather than analytically. Preclinical findings show that the combined interventions produce more pronounced effects on specific biomarkers such as BDNF, antioxidant enzyme levels, and mitochondrial regulators like PGC-1α. In cancer models, enhanced tumor suppression has been associated with increased apoptosis and reduced proliferation when polyphenols and exercise are administered together. However, dose–response relationships remain poorly defined. Some studies indicate greater benefits with higher polyphenol doses or more intense exercise, yet others show diminishing returns or adverse effects at high doses, especially in oxidative-sensitive tissues. Further research using factorial designs and interaction statistics is essential to determine the optimal combination strategies and validate true synergy. While evidence for the synergistic effects of polyphenols and exercise is growing, few studies provide visual or statistical representations, such as forest plots or subgroup meta-analyses, that compare the magnitude of effects between single and combined interventions. Such tools would be instrumental in quantifying the intensity of synergy and establishing clinical relevance. In addition, comparative data on specific combinations of polyphenols and exercise types remain limited. Preliminary findings suggest that resveratrol may potentiate mitochondrial biogenesis and neuroprotection when combined with endurance exercise, while curcumin appears more effective in modulating inflammatory and apoptotic pathways when paired with resistance training. However, these observations are based primarily on preclinical models. Systematic evaluations are needed to determine optimal pairings of polyphenol type, dose, and exercise modality using standardized outcome metrics and interaction statistics. While this review does not include original pharmacokinetic data, it is important to note that drug metabolism in preclinical models such as rats is typically assessed through ADME studies, which evaluate the absorption, distribution, metabolism, and excretion of compounds. Metabolites formed during drug digestion may vary in activity and toxicity, and all animal waste generated during such studies is treated as biohazardous material and disposed of according to institutional safety protocols.

### 6.1 Limitations and future directions

Despite the growing body of evidence supporting the synergistic effects of polyphenols and exercise on neuroprotection, tumor suppression, and metabolic regulation, several critical limitations hinder the clinical translation of these findings. Most notably, the majority of supporting studies are based on preclinical animal models, which do not fully capture the complexity of human physiology, lifestyle variability, or disease heterogeneity. Small sample sizes, short follow-up durations, and the absence of multicenter trials further weaken the strength of existing evidence. These limitations restrict our ability to generalize findings or draw firm conclusions about long-term clinical outcomes. A major barrier to clinical translation lies in the lack of standardized protocols for both polyphenol supplementation and exercise interventions. Doses, durations, and delivery mechanisms vary widely across studies, making comparisons difficult and impeding replication in clinical settings. Furthermore, polyphenol bioavailability in humans is often low and inconsistent, influenced by individual differences in metabolism, gut microbiota, and dietary context.

Another challenge is the limited integration of personalized medicine approaches. Most clinical studies do not stratify patients by genetic background, metabolic profiles, or pre-existing conditions—factors that significantly influence response to both diet and exercise. This heterogeneity dilutes statistical power and limits the development of targeted interventions. To overcome these barriers, future research should prioritize large-scale, multicenter trials with standardized intervention protocols and long-term follow-up. Incorporating multi-omics profiling and systems biology tools will also be essential to identify predictive biomarkers and personalize interventions. Addressing these gaps is critical to translating the promising synergistic effects of polyphenols and exercise into safe, effective, and accessible clinical therapies. Despite promising findings, current research on the combined effects of polyphenols and exercise in brain function, HNC therapy, and metabolic regulation is hindered by several methodological shortcomings. First, there is a notable scarcity of large-scale, randomized controlled trials in human populations. Most available studies are preclinical or small pilot trials, limiting the generalizability and clinical translation of the findings. Furthermore, interindividual variability in dietary habits, physical fitness, and genetic predispositions is rarely accounted for, underscoring the need for personalized and stratified study designs. Second, many animal studies rely on short-term or acute interventions, which may not accurately reflect chronic disease processes such as neurodegeneration or cancer progression. Longitudinal animal studies with extended follow-up periods and functional outcome measures are needed to better understand long-term therapeutic effects and safety profiles. Third, *in vitro* studies often utilize simplified monoculture systems that fail to mimic the complexity of human physiology, including the tumor microenvironment or neurovascular units. These limitations restrict mechanistic insight and translational potential. Advanced models such as organoids, co-culture systems, and microfluidic platforms offer more physiologically relevant alternatives and should be prioritized in future studies. Finally, there is limited integration of systems biology approaches. The application of multi-omics techniques, including genomics, transcriptomics, proteomics, metabolomics, and microbiome analysis, could help identify biomarkers of response and elucidate the molecular basis of exercise-polyphenol synergy. These tools are also essential for developing personalized intervention strategies based on individual molecular profiles. Addressing these methodological limitations through interdisciplinary, long-term, and large-scale studies will be critical for advancing our understanding and optimizing the therapeutic application of combined polyphenol and exercise interventions.
